# Transient Delivery of A-C/EBP Protein Perturbs Differentiation of 3T3-L1 Cells and Induces Preadipocyte Marker Genes

**DOI:** 10.3389/fmolb.2020.603168

**Published:** 2021-01-25

**Authors:** Nishtha Sharma, Raminder Kaur, Binduma Yadav, Koushik Shah, Harshita Pandey, Diksha Choudhary, Prateek Jain, Aanchal Aggarwal, Charles Vinson, Vikas Rishi

**Affiliations:** ^1^National Agri-Food Biotechnology Institute (NABI), Mohali, India; ^2^Department of Biotechnology, Panjab University, Chandigarh, India; ^3^Regional Centre for Biotechnology (RCB), Faridabad, India; ^4^National Cancer Institute, National Institutes of Health, Bethesda, MD, United States

**Keywords:** C/EBPβ, protein transfection, adipogenesis, 3T3-L1, dedifferentiation, obesity

## Abstract

Transformation of committed 3T3-L1 preadipocytes to lipid-laden adipocytes involves the timely appearance of numerous transcription factors (TFs); foremost among them, C/EBPβ is expressed during the early phases of differentiation. Here, we describe liposome-mediated protein transfection approach to rapidly downregulate C/EBPβ by A-C/EBP protein inhibitor. Signals from EGFP-tagged A-C/EBP protein were observed in 3T3-L1 cells within 2 h of transfections, whereas for A-C/EBP gene transfections, equivalent signals appeared in 48 h. Following transient transfections, the expression profiles of 24 marker genes belonging to pro- and anti-adipogenic, cell cycle, and preadipocyte pathways were analyzed. Expectedly, the mRNA and protein expression profiles of adipocyte marker genes showed lower expression in both A-C/EBP protein- and gene-transfected samples. Interestingly, for preadipocytes and cell fate determinant genes, striking differences were observed between A-C/EBP protein- and A-C/EBP gene-transfected samples. Preadipocyte differentiation factors *Stat5a* and *Creb* were downregulated in A-C/EBP protein samples. Five preadipocyte markers, namely, *Pdgfrα*, *Pdgfrβ*, *Ly6A, CD34*, and *Itgb1*, showed high expression in A-C/EBP protein samples, whereas only *Ly6A* and *CD34* were expressed in A-C/EBP gene-transfected samples. *Pdgfrα* and *Pdgfrβ*, two known cell fate markers, were expressed in A-C/EBP protein-transfected samples, suggesting a possible reversal of differentiation. Our study provides evidences for the immediate and efficient knockdown of C/EBPβ protein to understand time-dependent preadipocytes differentiation.

## Introduction

With an increase in severe obesity load worldwide and co-morbidities associated with it, there is an unprecedented interest in adipocyte biology. Adipogenesis is defined as the formation of lipid-laden adipocytes from mesenchymal stem cells. The terminal differentiation process, i.e., adipocytes formation from committed preadipocytes, is closely mimicked *ex vivo* by 3T3-L1 cell lines when induced with hormone cocktail (Green and Kehinde, 1974). Previous evidences have suggested the pivotal role of peroxisome proliferator-activated receptor gamma (PPARγ) and members of the CCAAT/enhancer-binding protein (C/EBP) family in the entire terminal differentiation process of adipogenesis (Farmer, 2006). C/EBPβ and C/EBPδ are first to express after induction by hormone cocktail and are known to direct the process of differentiation by transcriptionally activating promoters of *C/EBPα* and *PPARγ*, which further lead to the expression of adipocyte-specific genes and subsequent terminal differentiation (MacDougald and Lane, 1995; Ntambi and Kim, 2000). Knocking out *C/EBPα* and *C/EBPβ* result in impaired adipogenesis, whereas knocking down *C/EBPβ* by siRNA inhibited mitotic clonal expansion (MCE), a prerequisite for adipogenesis (Tanaka et al., 1997; Zhang et al., 2011). Targeting members of the C/EBP family of bZIP transcription factors (TFs) at the initial stages of adipogenesis may bring changes in the final morphology and metabolic state of adipocytes, or it may inhibit the differentiation process. Strategies used for inhibiting bZIPs, such as the use of small molecule inhibitors, siRNAs, and nucleases like CRISPR/Cas, though are effective but suffer from limitations of cytotoxicity, non-specificity, and off-target effects (Rishi et al., 2005; Payne et al., 2010; Lundh et al., 2017). Another strategy to functionally inactivate a gene is by over-producing its inhibitory variant, known as dominant-negative. C/EBPβ activity is regulated in the cells by C/EBP homologous protein-10 (CHOP-10), a natural dominant-negative that heterodimerizes with C/EBPβ, delays its DNA binding, and invigorates preadipocytes to two cycles of MCE, followed by the expression of anti-mitotic C/EBPα (Tang and Lane, 2000). A-C/EBP, a designed repressive protein of C/EBP, has N-terminal acidic extension appended to C/EBP leucine zipper. It forms stable heterodimer with wild-type C/EBPβ, preventing its DNA binding and entry into the nucleus, thus blocking MCE and inhibiting adipogenesis (Krylov et al., 1995; Zhang et al., 2004b). In addition, by preferentially heterodimerizing with target bZIP, A-C/EBP may preclude the interacting partners of C/EBPβ in the cell. Considering the ability of A-C/EBP to specifically inhibit C/EBPβ *in vivo*, we decided to use A-C/EBP protein and A-C/EBP plasmid DNA to decipher the time-dependent role of C/EBPβ and its interacting partners in preadipocytes differentiation.

As the process of adipogenesis involves spatial and temporal cascade of gene expression, the protocols using external DNA or RNA for transfections seem a bit moderate. Usually, the genetic methods of inhibiting the expression of a protein take 24–48 h, and during the time-frame, cellular and molecular requital may occur, blocking the expected phenotype (Fan et al., 2014). The expression and temporal levels of proteins are difficult to control with nucleic acid delivery strategies since amplification steps cannot be controlled accurately (Chiper et al., 2018). In addition, DNA-mediated protein overexpression perturbs the metabolic states of the cells by promoting non-specific interactions (Bhattacharyya et al., 2016). Protein delivery is independent of transcription and translation machinery of the cells and acts immediately unlike the gene expression from nucleic acids (Gaj and Liu, 2015). Protein transfection titration experiments may delineate specific interaction from non-specific interactions, can be used as a tool for basic research, and may have therapeutic applications. In this study, we hypothesized that during the early wave of adipogenesis if C/EBP's activity is inhibited in an immediate and timely manner during the ongoing differentiation process, it may lead to changes in the original morphology and metabolic state of the cells. Inhibition of C/EBPβ may affect the expression of its interacting partners and the genes they regulate during adipogenesis. It is known that the expression of C/EBPβ takes place within few hours of induction; therefore, direct delivery of inhibitor can sequester C/EBPβ and bring changes in the differentiation process.

## Materials and Methods

### Cell Culture and Differentiation

3T3-L1 cell lines were obtained from NCCS, Pune (India) at passage number 16. The cells were cultured and maintained in Dulbecco's Modified Eagle's Medium (DMEM) supplemented with 10% (vol/vol) bovine serum, 100 U/ml penicillin, and 100 μg/ml streptomycin, in 5% CO_2_ incubator with 98% humidity. The cells were grown till 60–70% confluency before differentiation and transfection experiments. The cells were differentiated on day 0 with differentiation media containing DMEM supplemented with 10% fetal bovine serum (FBS) and MDI hormone cocktail (0.5 mM 3-isobutyl-1-methylxanthine, 1 μM dexamethasone, and 1 μg/ml insulin) for 48 h. After 48 h, differentiation media was replaced with adipocyte maintenance media containing DMEM supplemented with 10% FBS and 1 μg/ml insulin. After day 4, the cells were fed every other day with DMEM supplemented with 10% FBS. The cells were seeded in 60 mm dishes for transfection and differentiation experiments. Transfected cells were collected post-differentiation for RNA and protein isolation and subjected to mRNA expression analysis and Western blotting experiments.

### Cloning, Protein Expression, and Purification

Cloning of dominant-negative A-ZIPs, i.e., A-C/EBP and A-VBP in pT5 bacterial expression system, has been described earlier (Rishi et al., 2004). A-ZIPs (A-C/EBP and A-VBP) were cloned as KpnI-EcoRI fragments into pCMV, a mammalian vector (Matsuda, 2015). For localization studies, EGFP was cloned as HindIII-NotI fragment immediately downstream of A-C/EBP in pT5 vector. Vectors containing dominant-negative genes were used to transform *Escherichia coli* (BL21 DE3), grown overnight at 37°C in Super Broth containing 100 μg/ml ampicillin and 35 μg/ml chloramphenicol. Then, 20 ml primary cultures were transferred to 500 ml Super Broth containing 100 μg/ml ampicillin and grown until OD reached 0.6. Cultures were then induced with 1 mM isopropyl-β-D-thiogalactopyranoside (IPTG), harvested after 3 h, and processed as described earlier (Krylov et al., 1994). Dialysis was performed against low salt buffer [20 mM Tris–HCl pH 8.0, 50 mM KCl, 1 mM ethylenediaminetetraacetic acid (EDTA), 0.2 mM phenylmethylsulfonyl fluoride (PMSF), and 1 mM dithiothreitol (DTT)]. Dialyzed A-C/EBP and A-VBP proteins were passed through a hydroxyapatite column and eluted with dialysis buffer containing 250 mM sodium phosphate buffer (pH 7.4). Proteins were further purified to high homogeneity using reverse phase analytical HPLC system equipped with C-18 hydrophobic column as described earlier (Krylov et al., 1994). Lyophilized HPLC purified proteins were dissolved in phosphate buffer saline (PBS) with EDTA and resolved on SDS-PAGE to check the purity and quality (Supplementary Figure 1A). Proteins were quantified using UV spectroscopy, and concentrations were calculated according to a previously published method (Krylov et al., 1994). IPTG induced and uninduced samples of EGFP and A-C/EBP-EHFP were checked on SDS-PAGE. A-C/EBP-EGFP was also purified as described above (Supplementary Figure 1B). Samples were dialyzed, and purified protein was used for transfections.

### Liposome-Mediated Cell Transfections and Confocal Microscopy

Two days prior transfections, the cells were seeded in 6-well-plates/60 mm dishes. For plasmid transfections, 2.5 μg of plasmid DNA was added to 50 μl low serum media (LSM) containing DMEM media and 2% serum without antibiotics, followed by the addition of 5 μl of Lipofectamine 2000 in 50 μl LSM. The plasmid and Lipofectamine mixture was incubated for 15–20 min at room temperature (RT) and then added to the 3T3-L1 cells. MTT [3-(4,5-dimethylthiazol-2-yl)-2,5-diphenyltetrazolium bromide] assays were performed to check cell viability at different concentrations of A-C/EBP protein (0.1–20 μM). For A-C/EBP protein transfections, pure protein was added to 50 μl LSM so that the final concentration was 3 μM, followed by the addition of 50 μl LSM containing 5 μl of Lipofectamine 2000. After incubation for 20 min at RT, the mixture was added to the cells. The workflow for protein and gene transfections is given in the supplementary section (Supplementary Figure 2A). It shows the transfection experiments with A-C/EBP protein and A-C/EBP gene (Supplementary Figure 2A). For experiments with A-C/EBP gene, two conditions were used. (1) 3T3-L1 cells were transfected with CMV:A-C/EBP plasmids for 2 h, followed by differentiation, and (2) the cells were transfected with CMV:A-C/EBP plasmids for 48 h and subjected to differentiation protocol. The first condition was to mimic the timing of protein transfections (2–6 h) and aims to compare the temporal effect of protein and gene transfections, since protein manifests immediately after transfection, whereas a gene takes ~48 h to transcribe and translate. Similarly, A-C/EBP-EGFP protein was used to transfect the cells on a culture slide, and after 2 h, the cells were fixed and treated with Hoechst stain. In addition, A-C/EBP (30–40 μg) protein was labeled with Alexa Fluor 488 according to the manufacturer's protocol (Thermo Fisher). The time-lagged expression of EGFP-tagged A-C/EBP protein was measured after transfecting the cells with CMV:A-C/EBP-EGFP plasmid for 2 and 48 h. Fluorescence signals from the fixed cells were visualized and photographed using a confocal microscope (Carl-Zeiss LSM 880).

### Oil Red O Staining

For Oil Red O (ORO) staining, 3T3-L1 cells were washed three times with PBS and fixed for 20 min using 10% (final concentration) formaldehyde. ORO stain (0.5% in isopropanol) was diluted with water (3:2) and filtered through a 0.45 μm filter. The cells were incubated with filtered ORO for 1 h at RT. Stained cells were washed 2–3 times with water, visualized by light microscopy, and photographed. ORO-stained cells were eluted in 100% isopropanol, and signals were quantified by taking absorbance at 520 nm. Neat isopropanol was used as blank.

### Protein Estimation

The protein contents were estimated by the bicinchoninic acid (BCA) method using a Puregene BCA protein assay kit.

### Immunoblotting

A-C/EBP, A-C/EBP-EGFP, and A-VBP proteins expressed in bacteria and 3T3-L1 cells have 13 amino acids long T7 tag at the N-terminal that was used for immunodetection. Cell monolayers (6 cm dishes) at different time points were washed with cold PBS, pH 7.4, and then scraped in the presence of RIPA buffer (150 mM NaCl, 0.1% Triton X-100, 0.5% sodium deoxycholate, 0.1% SDS, 50 mM Tris–HCl pH 8.0, and protease inhibitors). Cell lysates were cleared by centrifugation, and supernatants were heated at 98°C for 10–12 min.

Samples with an equal amount of total protein (30 μg) were separated by 15% SDS/PAGE. Pre-stained protein ladder was run in parallel alongside samples. Gel-resolved proteins were transferred to polyvinylidene fluoride (PVDF) membranes in cool conditions for 2 h in transfer buffer (25 mM Tris, 190 mM glycine, 20% methanol, and pH 8.3). The membrane was blocked using 3% bovine serum albumin (BSA) in Tris-buffered saline (TBS) containing 0.1% Tween 20 for 1 h at RT. T7-tag HRP-conjugated antibody (Thermo Fisher, Cat. no. PA1-31449) was used at a dilution of 1:10,000 overnight at 4°C, and the membrane was probed for protein using an ECL chemiluminescence kit (BioRad). HPLC purified protein was used as positive control. An identical parallel blot was run and probed for β-actin and used as a loading control (Cell Signaling Technologies, Cat. no. 4967S). Immunodetection of A-C/EBP-EGFP was performed from samples generated from transfected cells using T7-tag HRP-conjugated primary antibody (Thermo Fisher, Cat. no. PA1-31449). For time-course experiments involving A-C/EBP protein, eight time periods (1–6, 8, and 10 h) samples were collected, whereas for A-C/EBP plasmid, six time periods (0, 6, 8, 24, 48, and 72 h) samples were probed for A-C/EBP signals. The combined intensities of monomer and dimer forms were used for quantification and graphical representations.

For C/EBPβ, anti-C/EBPβ primary antibody (Biolegend, Cat. no. 606202) was used at a 1:2,000 dilution overnight at 4°C. Blots were washed with Tris-buffered saline with Tween 20 (TBST) and incubated with anti-mouse (Cell Signaling Technologies, Cat. no. 7076P2) HRP-conjugated secondary antibody for 2 h at RT. For C/EBPδ, anti-C/EBPδ antibody (Cloud-Clone, Cat. no. PAC462Mu01) was used overnight at a 1:1,000 dilution. For adipocyte marker genes, protein was isolated from transfected cells after 4 days differentiation. Anti-C/EBPα (Cell Signaling Technologies, Cat. no. 8178S), anti-PPARγ (Cloud-Clone, Cat. no. PAA886Mu01), and anti-aP2 (Cloud-Clone, Cat. no. PAB693Mu01) were used at dilutions of 1:2,000, 1:1,000, and 1:1,000, respectively, with overnight incubation. Blots were probed with anti-rabbit (Cell Signaling Technologies, Cat. no. 7074P2) secondary antibody for 2 h at RT. β-Actin was used as loading control. Proteins were visualized by the ECL chemiluminescence method using an Amersham imager 600 (GE Healthcare), and densitometry of protein bands was performed with the NIH ImageJ software.

### RNA Isolation, qPCR, Protein–Protein Interaction Network Prediction, and Flow Cytometry

For gene expression study, transfected cells were lysed with TriZol reagent, and total RNA was isolated as per the manufacturer's instructions. Isolated total RNA was treated with DNaseI to remove traces of genomic DNA. Then, 1 μg of total RNA was reverse transcribed to cDNA in a final reaction volume of 20 μl using a BioRad iScript cDNA synthesis kit. Quantitative PCR (qPCR) was performed with gene-specific primers (Supplementary Table 1) using iTaq universal SYBR Green Supermix (BioRad) for 45 cycles on CFX96 Real Time System (BioRad). The C_t_ values obtained were normalized against β-actin because of its consistent expression as observed in Western blots. qPCR data were analyzed by the comparative C_t_ method (Schmittgen and Livak, 2008). The factors involved in adipogenesis and MCE were selected from the literature (Zhang et al., 2004b; Jiang et al., 2012). Selected factors were put in the STRING database in the Multiple protein sections by names/identifiers section, and interaction data was retrieved. For preadipocyte marker genes, RNA was isolated from transfected cells 5 days post-differentiation, and qPCR was performed using gene-specific primers retrieved from Harvard Primer Bank (Supplementary Table 3).

For cell cycle analysis, A-C/EBP protein- and gene-transfected cells were induced with differentiation media till the initiation of MCE, i.e., around 18 h after induction. After induction, the cells were fixed with 70% ethanol for 30 min and centrifuged for 5 min at 1,300 rpm. The cells were washed twice with FACS buffer containing PBS and 0.3% BSA. Propidium iodide (PI) staining solution containing PBS, PI (50 μg/ml), RNaseA (0.1 mg/ml), and 2 mM magnesium chloride (MgCl_2_) was added to the washed pellet, and the cells were incubated at RT for 20 min. Flow cytometry was performed based on an earlier study (Kim and Sederstrom, 2015).

### Chromatin Immunoprecipitation Analysis

For analyzing the binding of C/EBPβ to the promoters of adipogenic genes, ChIP assay was performed as described earlier with minor modifications (Massie and Mills, 2012). Briefly, the cells transfected with gene and protein along with untreated cells were cross-linked with 1% formaldehyde for 15 min at RT. The cross-linking reaction was stopped with the addition of 0.125 M glycine for 5 min. The cells were washed with ice-cold PBS containing protease-inhibitor cocktail (PIC) and centrifuged at 3,000 rpm for 5 min. The pellet was suspended in lysis buffer [50 mM HEPES–KOH (pH 7.5), 140 mM NaCl, 1 mM EDTA pH 8, 1% Triton X-100, 0.1% sodium deoxycholate, 0.1% SDS, and PIC] and kept on ice for 10 min. The suspended cells were sonicated for 8 cycles (30 s on and 30 s off) with 30% amplitude on ice using a probe sonicator (Sonics Vibra Cells; Fisher Scientific), followed by centrifugation at 9,000 rpm for 10 min. RNA and protein-free DNA samples were obtained by adding RNase and proteinase K to 50 μl of sample at 65°C for 2 h and purifying the DNA using a PCR purification kit (Thermo Fisher Scientific). Purified DNA was run on 1.5% agarose gel to check the efficiency of chromatin shearing. Then, 2 μg of chromatin was used for immunoprecipitation (IP). Samples were incubated at 4°C overnight with anti-C/EBPβ and non-specific rabbit IgG. The sample without IP was used as input control. On the next day, protein G beads (G Biosciences) were vortexed and added to antibody–chromatin samples for 2 h at 4°C with rotation. Beads were pelleted down using a magnetic separation rack (NEB, USA) and washed with low salt buffer, followed by high salt buffer and LiCl buffer for 5 min each at 4°C. Elution buffer (1% SDS, 100 mM NaHCO_3_) was added to the beads and input samples and incubated for 30 min at 65°C. Eluted chromatin was reverse cross-linked by adding 5 M NaCl and proteinase K for 2 h at 65°C. Samples were passed through a spin column, and eluted DNA was used for semi-quantitative and qPCR for 32–40 cycles with specific primers (Supplementary Table 2) (Tang et al., 2004). PPARγ *Mus musculus*, transcript variant 2, mRNA has been used for primer designing in a previous study for ChIP-PCR. The same primers have been used in a previous study for ChIP-PCR of C/EBPα, PPARγ, and 422/aP2 (Tang et al., 2004). Analysis of qPCR results was done by using the fold enrichment method [(Fold enrichment = 2^−ddCt^), where ddCt = Ct (IP) – Ct (IgG)].

### Statistical Analysis

For gene expression analysis, means, and standard errors were calculated from six biological replicates each with three replicates. The statistical significance was determined by Student's *t*-test. For Western blotting, means and standard errors were calculated from three biological replicates. The statistical significance was determined by one-way ANOVA. A difference in *p*-value of <0.05 is considered as significant.

## Results

### Cellular Uptake of A-C/EBP Protein and Its Impact on Lipid Accumulation of Murine 3T3-L1 Cells

Before embarking on differentiation experiments, the cytotoxic effect of A-C/EBP protein was tested on 3T3-L1 cells by performing MTT assay in a concentration range 0.1–20 μM (Supplementary Figure 1C). Percentage survival at 3 μM was 80%, confirming the non-toxicity of A-C/EBP.

**Figure 1:**
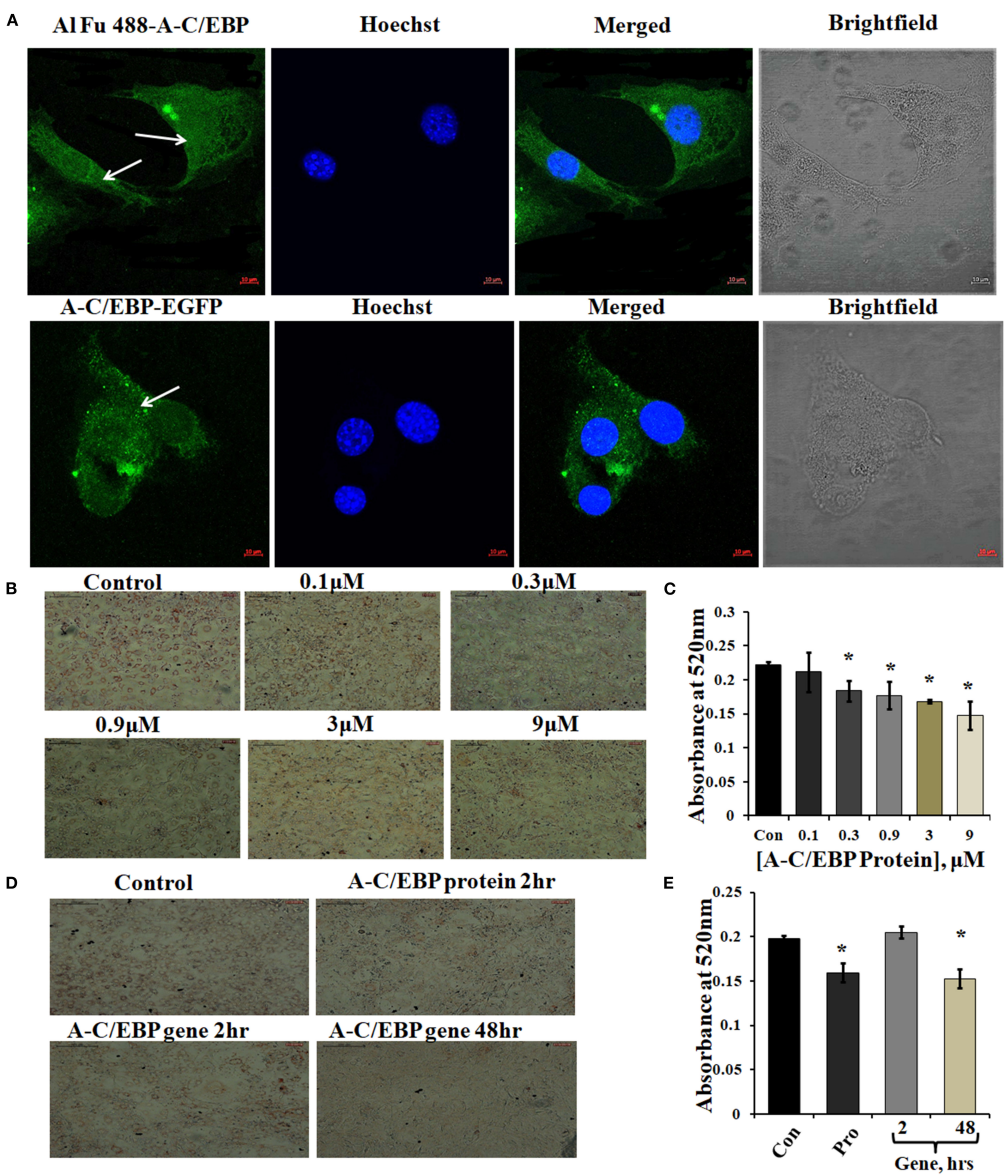
Localization of protein in the cytoplasm and ORO staining of transfected and differentiated cells. **(A)** Image representing Alexa Fluor (Al Fu) 488 and EGFP-tagged protein inside the cytoplasm; arrows depict the presence of tagged A-C/EBP in the cytoplasm, counterstained with Hoechst 33342; magnification = 40×, scale bar = 10 μm. **(B)** Representation of ORO-stained cells transfected with A-C/EBP protein at different concentrations along with untreated cells. Half-log concentrations of A-C/EBP protein were taken. Magnification = 10×, scale bar = 200 μm. **(C)** Quantification of ORO-stained samples treated with A-C/EBP protein. Absorbance was taken at 520 nm, and isopropanol was taken as blank. **(D)** Image representing ORO staining of cells transfected with A-C/EBP protein and gene; magnification = 10×, scale bar = 200 μm. **(E)** Quantification of ORO-stained samples treated with A-C/EBP protein and gene, absorbance was taken at 520 nm, and isopropanol was taken as blank. ^*^Significantly different from control (*p* < 0.05).

Three experimental conditions were used to observe the time-dependent appearance of fluorescent tagged A-C/EBP protein signals in 3T3-L1 cells: (1) cells transfected with A-C/EBP protein for 2 h, (2) A-C/EBP plasmid transfected for 2 h, and (3) cells transfected with A-C/EBP plasmid for 48 h. Confocal microscopy was used to demonstrate the cellular uptake and localization of A-C/EBP in 3T3-L1 cells. Post-transfections, in protein samples, fluorescence signals were detected within 2 h. For studies involving A-C/EBP plasmid, we followed the earlier reported gene-delivery protocols that considered requirement of 48 h for the gene to transcribe and translate. Both Alexa Fluor 488 (Al Fu 488) tagged A-C/EBP and A-C/EBP-EGFP (37 kDa) proteins were internalized by the cells, and intense A/CEBP protein signals were observed within 2 h (Figure 1A). Arrows indicate the presence of tagged A-C/EBP in the cytoplasm of 3T3-L1 cells. The cells transfected with CMV:A-C/EBP-EGFP plasmid showed low EGFP expression in 2 h that intensified in 48 h and became equivalent to 2 h protein samples (Supplementary Figure 3A). Immunoblotting of A-C/EBP-EGFP from transfected cells showed a similar trend as observed in microscopy experiments (Supplementary Figure 3B).

A-C/EBP is negatively charged and highly anionic protein, this may facilitate its entry in the cell by the same electrostatic-driven complexation as seen for liposome-nucleic acids (Zuris et al., 2015). Our results show that liposomes can carry and deliver large anionic protein A-C/EBP-EGFP (37 kDa) inside the cells. We further determined the impact of A-C/EBP on the morphology of 3T3-L1 cells after differentiation. An equal number of cells were seeded in 6-well plates (30,000 cells/well). Then, 60–70% confluent preadipocytes were treated with increasing concentrations of A-C/EBP ranging from 0.1 to 9 μM. Ten days post-differentiation, the cells showed lower lipid accumulation with increasing concentration of A-C/EBP as evidenced by ORO staining compared with untransfected control cells (Figure 1B). Absorbances were normalized to the background absorbance. Quantification of ORO-stained cells showed a linear decrease in lipid accumulation with increasing concentrations of A-C/EBP (Figure 1C). In another set of experiments, 3T3-L1 cells treated with A-C/EBP protein (3 μM) for 2 h and A-C/EBP plasmid (1 μg) for 48 h led to an equivalent decrease in lipid accumulation (Figures 1D,E). The cells transfected with A-C/EBP plasmid showed similar reduced differentiation status as protein transfections only after a lag of 2 days, suggesting a time lag required to transcribe and translate A-C/EBP protein. Compared with untransfected control cells (after 8 days of differentiation), the cells transfected with A-C/EBP protein for 2, 4, and 6 h showed reduced lipid accumulation (Supplementary Figure 1D). Transfection with A-VBP (Krylov et al., 1995), a dominant-negative inhibitor of VBP (bZIP TF that does not play a role in adipogenesis), did not affect lipid accumulation (Supplementary Figure 2B). ORO staining of A-C/EBP protein-transfected cells after 2 days of differentiation showed non-significant difference compared with control (Supplementary Figure 3C).

### Time-Dependent Appearance of A-C/EBP Protein in the Cells and Lower Expression of Adipocyte-Specific Markers in Instilled Cells

**Figure 2:**
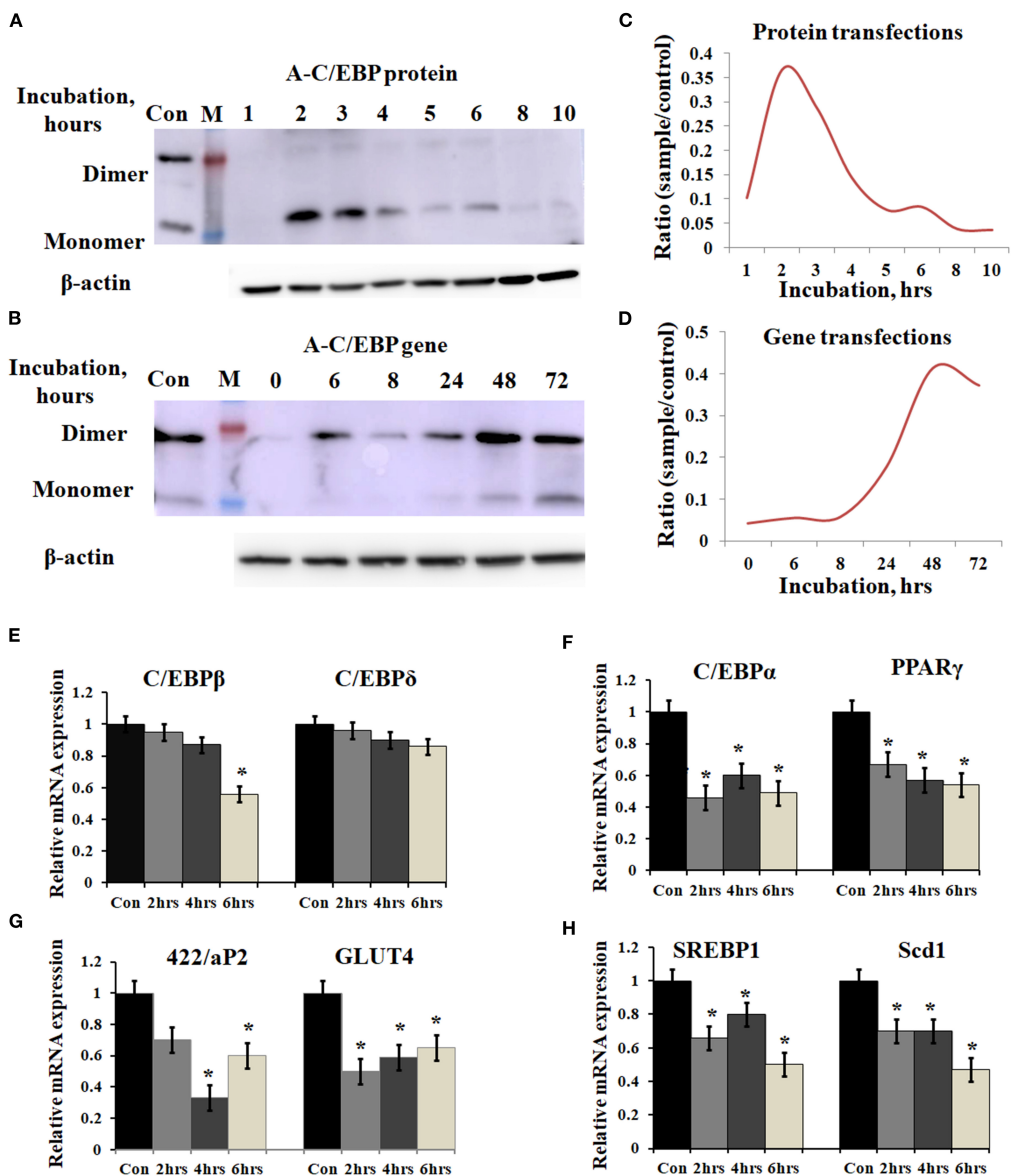
Time-dependent presence of A-C/EBP protein and A-C/EBP gene products in 3T3-L1 cells and effect of A-C/EBP protein on adipocyte marker genes. **(A)** Western blots show the signals of A-C/EBP protein when the cells were treated with liposome-entrapped A-C/EBP protein. With time, signal intensity decreased due to degradation of A-C/EBP protein. Pure protein was used as control. **(B)** Western blot shows the time-dependent profile of A-C/EBP gene product. Here, the signal intensity increased and peaked at 48 h and remained so at 72 h. Pure protein was used as control. **(C,D)** Curves show A-C/EBP signal trends plotted from Western blot experiments over indicated time. Opposite trends were obtained in protein- and gene-transfected cells. ^*^Significantly different from the control group (*p* < 0.05). Values are expressed as mean ± SD; *n* = 3 independent experiments. **(E–H)** Real-time PCR analysis of C/EBPs and *PPAR*γ, *422/aP2, GLUT4, SREBP1*, and *Scd1* w.r.t β-actin in cells transfected with A-C/EBP for 2, 4, and 6 h before differentiation. ^*^Significantly different from control (*p* < 0.05). Values are expressed as mean ± SD; *n* = 6 independent experiments.

3T3-L1 cells were transfected with A-C/EBP protein and gene for different time intervals, as shown in Figures 2A,B. Western blots analysis showed A-C/EBP gene expression after 24 h, peaked at 48 h, and persisted till 72 h (Figure 2B), whereas the levels of purified A-C/EBP protein delivered directly into the cells, peaked in just 2 h of transfection, then decreased rapidly, and were barely detectable after 10 h (Figure 2A). Although a low expression of A-C/EBP protein was also observed in 6 h gene-transfected samples, equivalent signals were observed only after 48 h of gene transfections. The trend lines showed change in the expression of A-C/EBP in protein- and gene-transfected cells at different time intervals (Figures 2C,D). In solution, A-C/EBP dimer exists in equilibrium with monomer ([D]⇔[M].[M]). In protein-transfected samples where low concentrations of A-C/EBP were used, A-C/EBP exists in monomeric form (Figure 2A). In gene-transfected samples, the use of strong viral (CMV) promoter resulted in the unregulated overexpression of A-C/EBP protein; therefore, dimer conformation dominates (Figure 2B). In gene-transfected samples, unknown post-translational modifications may also encourage dimer form.

Prompted by immunoblotting experiments, the cells were transfected with A-C/EBP protein for 2, 4, and 6 h and then differentiated with MDI hormone cocktail. RNAs were isolated from these samples, and qPCR was performed for *C/EBPβ*, *C/EBPδ*, and adipocyte marker genes (*C/EBPα*, *PPARγ*, *422/ap2, GLUT4, SREBP1*, and *Scd1*). Results showed no significant change in the gene expression of *C/EBPβ* and *C/EBPδ* within 2 and 4 h of transfected and differentiated cells (Figure 2E). At 6 h, a lower expression of *C/EBPβ* was observed. C/EBPβ autoregulates its own transcription by binding to its promoter region located between −121 and −71 (Niehof et al., 2001). We observed that the *C/EBPβ* expression was not affected by A-C/EBP protein in 4 h, but the gene expression decreased in 6 h suggesting the degradation of C/EBPβ**|**A-C/EBP heterodimer and the inhibition of the DNA-binding activity of C/EBPβ and its autoregulation. The mRNA expressions of six adipocyte-marker genes, i.e., *C/EBPα*, *PPARγ*, *422/ap2, Glut4, SREBP1*, and *Scd1*, were lower in all three experimental conditions of A-C/EBP protein transfections (Figures 2F–H).

**Figure 3:**
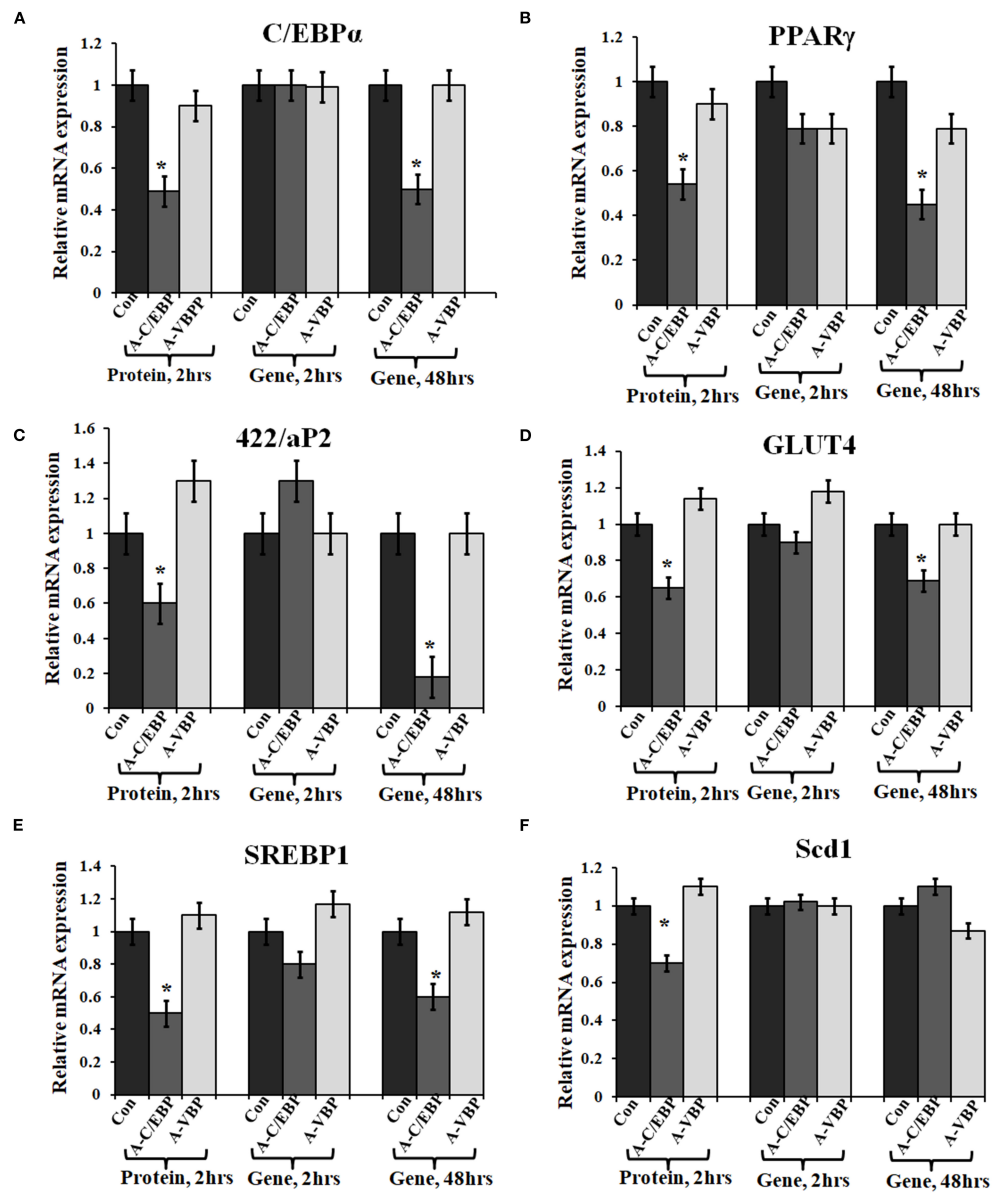
Effect of A-C/EBP and A-VBP protein and gene on six adipocyte marker genes. **(A–F)**. Gene expression analysis for comparative expression of *C/EBPα*, *PPAR*γ, *422/aP2, GLUT4, SREBP1*, and *Scd1* w.r.t. actin expression. 3T3-L1 cells were transfected with A-C/EBP and A-VBP protein. A-VBP is a designed dominant-negative of VBP transcription factor and used as a negative control here. All six marker genes show downregulation in cell samples transfected with 3 μM A-C/EBP protein, whereas only 48 h gene samples showed significant decrease. Since neither A-VBP protein nor its gene has any effect on the expression of six adipocyte marker genes, we conclude that A-C/EBP is specific in inhibiting preadipocyte 3T3-L1 cells differentiation. ^*^Indicate significantly different from control (*p* < 0.05). Values are expressed as mean ± SD; *n* = 6 independent experiments.

Primary embryonic fibroblasts from double knockout *C/EBPβ* (–/–) and *δ* (–/–) mice neither differentiate nor express the late TFs, i.e., C/EBPα and PPARγ (Zhang et al., 2011). Knocking down *C/EBPβ* with *A-C/EBP* has resulted in impaired adipogenesis, prevents MCE, and inhibits the expression of adipocyte marker *422/aP2* along with *C/EBPα* and *PPARγ* (Zhang et al., 2004b). We also observed a lower gene expression of selected adipocyte marker genes in A-C/EBP protein- as well as gene- (48 h) transfected cells, supporting the above observations. No significant difference in the expressions of these genes was observed in A-C/EBP gene transfection for 2 h. mRNA expression analysis of adipocyte marker genes from cells transfected with negative-control A-VBP protein and gene showed no significant change in the expression of adipocyte marker genes compared with untreated samples under three experimental conditions used (Figures 3A–F).

### Alterations in Adipogenic-Specific TFs and Preadipocyte Marker Genes in Transfected and Differentiated 3T3-L1 Cells

Taking cues from previous studies, we chose the foremost TFs involved in adipocyte differentiation (Jiang et al., 2012). In previous studies, differentiation factors are categorized into two groups responsible for the early wave and second wave of adipogenesis. The early wave of adipogenesis is initiated by factors that appear from induction till the 2nd day of differentiation. C/EBPβ is a key factor of this group as it appears within 2 h of induction with MDI treatment. Many other factors regulating the process of preadipocyte differentiation are present during the early wave. We have selected these TFs for their possible interactions with C/EBPβ, since targeting C/EBPβ may affect the expression of the selected TFs and genes regulated by them. Supplementary Figure 4A shows the putative interaction of C/EBPβ with the different TFs. For this study, the TFs chosen for gene expression analysis are both promoters and inhibitors of adipogenesis *KLF6, Stat5a, Zfp423, Zfp467, TCF7L1, Creb*, and *VEGF* are promoters of adipogenesis (Rosen and MacDougald, 2006; Cristancho et al., 2011; Lu et al., 2012; Wei et al., 2013), whereas *Pref1 (Dlk1), KLF2, GATA2/3*, and *FoxC2* are known to inhibit differentiation (Rosen and MacDougald, 2006). Among all the selected factors of the early wave of adipogenesis, *Stat5a, Creb, GATA2/3, FoxC2, KLF2, VEGF*, and *Cdk* are known to interact with *C/EBPβ* directly or indirectly (Shuman et al., 2004; Zhang et al., 2004a; Tong et al., 2005; Kang et al., 2013; Gan et al., 2016; Shinagawa et al., 2019). STRING database was used to demonstrate the interacting partners of *C/EBPβ* among the chosen TFs. *Stat5a, Creb*, and *Gata2/3* interact directly with *C/EBPβ* (Supplementary Figure 4A). Five TFs showed no interaction with *C/EBPβ*.

To quantify the effect of A-C/EBP protein and gene on the expression of major TFs involved in 3T3-L1 differentiation, the cells were treated with both purified A-C/EBP protein and *A-C/EBP* coding plasmid. A-VBP was used as negative control. mRNA samples from A-C/EBP-untreated and -treated cells were generated after 2 days of MDI induction. Then, 24 adipogenic-specific genes and other TFs involved in early adipogenesis were subjected to qPCR analysis. At 6 h post-transfection, the expression of *C/EBPβ* was significantly lower in cells transfected with A-C/EBP protein than in control and *A-C/EBP* gene transfections. *C/EBPδ* expression did not change significantly. Results demonstrate the high specificity of A-C/EBP in inhibiting C/EBPβ. Among pro-adipogenic genes, the expressions of *KLF6, Stat5a, Zfp467*, and *Creb* were lower in protein-transfected cells with no change in A-C/EBP gene-transfected cells (48 h). Among anti-adipogenic proteins, only *GATA2* showed higher expression in protein-transfected cells, whereas in gene-transfected cells (48 h), the expressions of all anti-adipogenic genes except *GATA2* were higher.

**Figure 4:**
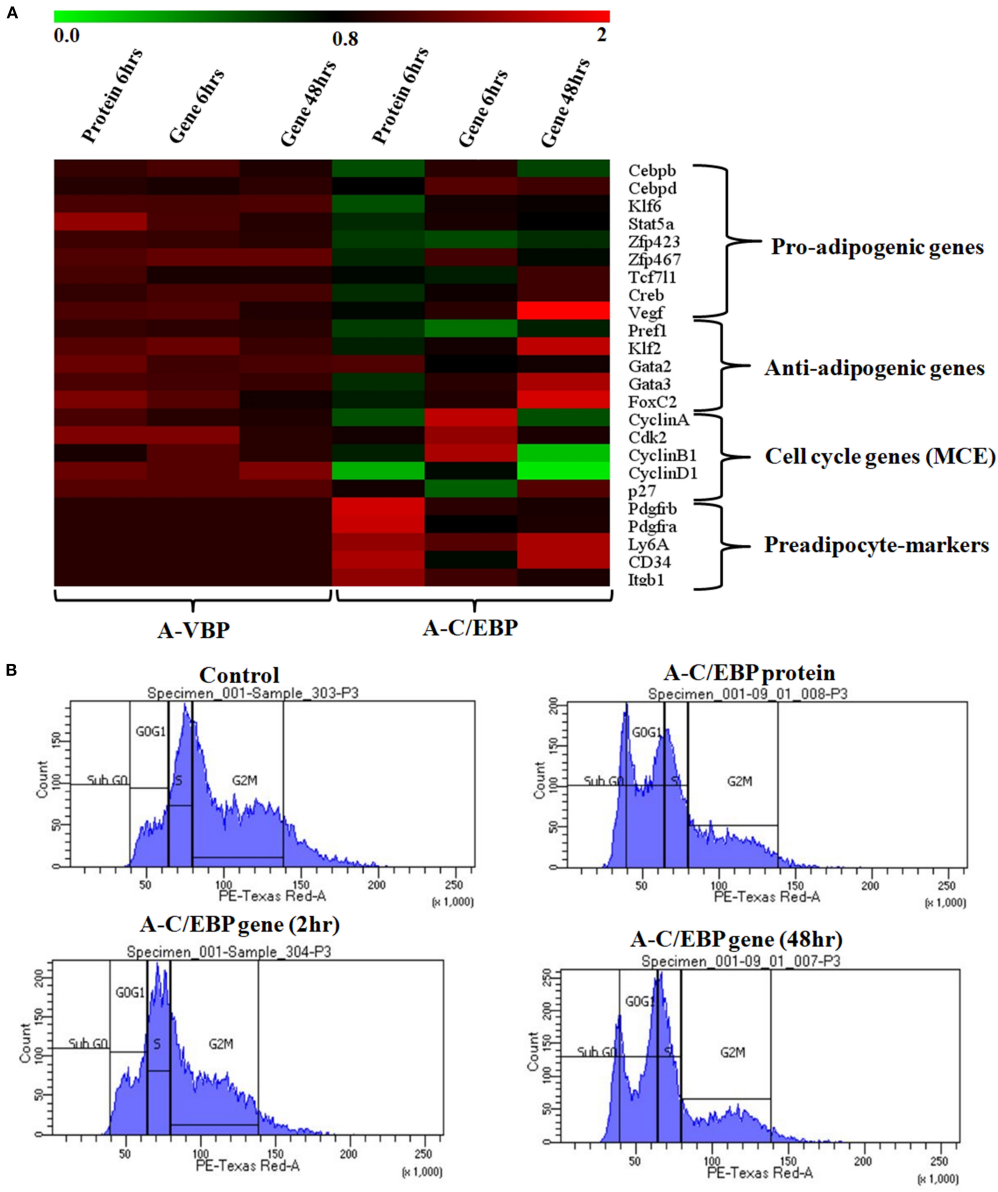
Effects of A-C/EBP and A-VBP protein and gene transfections at the indicated conditions on the expression of factors involved in adipogenesis, MCE, and preadipocyte state of 3T3-L1 cells and flow cytometry analysis. **(A)** Heat map representing real-time PCR analyses of the expression of 24 genes involved in the process of differentiation, MCE, and preadipocyte markers from the cells transfected with A-C/EBP and A-VBP protein and gene. The color indicates the fold-change value converted to log2 scale, as compared with untreated sample. The data represent the mean of six independent biological experiments, and transcript was normalized to actin. **(B)** Effect of A-C/EBP on change in cell cycle stages of transfected and differentiated cells analyzed by flow cytometry and plotted on a graph.

The expressions of selected cell cycle markers, i.e., *cyclin A, cyclin B1*, and *cyclin D1*, were lower in A-C/EBP protein and gene 48 h post-transfection samples, and the expression of *p27* was higher than control (Figure 4A). The degradation of p27 is necessary for cell cycle to progress (Zhang et al., 2004b). The high expression of *p27* and the low expression of cyclins suggest the inability of preadipocytes to undergo MCE even after induction. Inhibition of differentiation may be due to the prevention of C/EBPβ to localize into the nucleus after it heterodimerized with A-C/EBP (Lane et al., 1999; Zhang et al., 2004b). In 6 h gene-transfected cells, the expressions of *KLF6, Stat5a, Zfp423, Creb, KLF2, GATA2, GATA3, FoxC2*, and *VEGF* remain unchanged, but the expressions of *Pref1* and *p27* were lower with the higher expression of cell cycle genes. A-VBP-transfected cells showed no significant changes in mRNA expressions for all genes studied here under four experimental conditions (control, protein-transfected, gene 6 h post-transfection, and gene 48 h post-transfection) (Figure 4A).

To observe the effects of A-C/EBP on MCE, flow cytometry of transfected cells stained with propidium iodide was performed. Untreated cells and cells transfected with A-C/EBP gene for 2 h showed around 50 and 45% of the cells in the G2–M phase, respectively (Figure 4B, Supplementary Figure 4B). In A-C/EBP protein- and gene- (48 h) treated cells, around 37 and 36% of the cells were observed in the G0–G1 phase, respectively (Figure 4B, Supplementary Figure 4B). These results support our observation of higher *p27* expression in A-C/EBP-treated cells (Figure 4A), as its degradation is required for G1–S transition of the cells to enter MCE (Zhang et al., 2004b).

Based on the morphology of ORO-stained cells and lower lipid accumulation in A-C/EBP protein- and gene- (48 h) transfected cells, we performed qPCR for common preadipocyte marker genes (Cawthorn et al., 2012; Hepler et al., 2017; Wang et al., 2018). Among these markers, *Ly6A, CD34*, and *Itgb1 (CD29)* are common mesenchymal stem cell markers (Hepler et al., 2017). Signaling balance between *Pdgfrα/Pdgfrβ* modulates the progenitor cells to commit to either white (*Pdgfrβ*) or beige (*Pdgfrα*) adipocytes with very different metabolic profiles (Gao et al., 2018). Analysis of qPCR results showed an increase in the expression of all five common preadipocyte marker genes, i.e., *Pdgfrβ*, *Pdgfrα*, *Ly6A, CD34*, and *Itgb1* in A-C/EBP protein-transfected cells (Figure 4A). Surprisingly, in 48 h A-C/EBP gene-transfected cells, though the expression of marker genes *Pdgfrβ*, *Pdgfrα*, and *Itgb1* did not change significantly from control, *Ly6a* and *CD34* were found to be upregulated (Figure 4A). Of all five selected markers, *CD34* was expressed in both preadipocytes and adipocytes, whereas the rest of the four were expressed only in committed preadipocytes (Cawthorn et al., 2012).

### Effect of A-C/EBP on Protein Expression of C/EBPs and Adipocyte Markers

**Figure 5:**
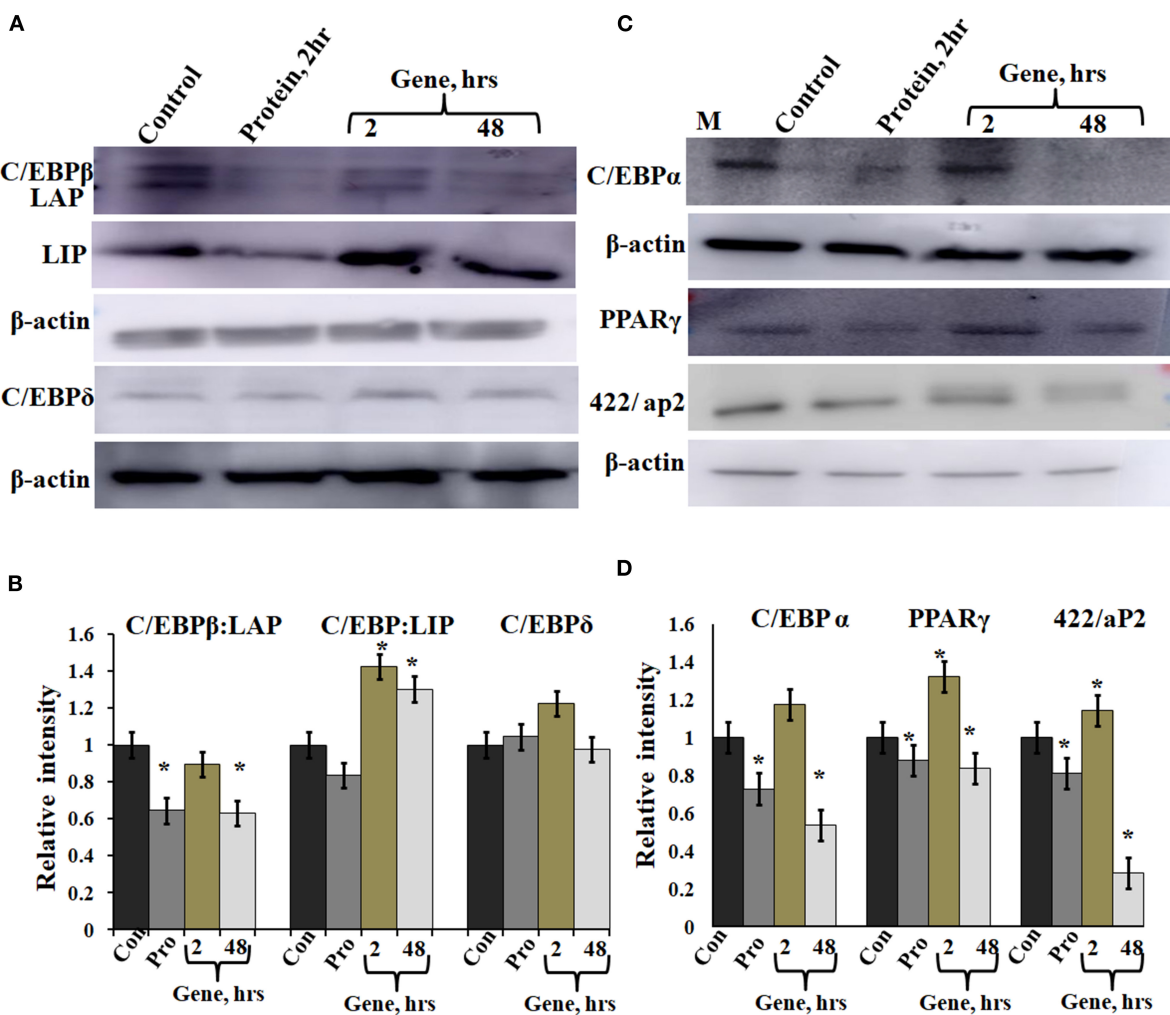
Expression of C/EBPβ, C/EBPδ, and adipocyte marker genes in cells transfected with A-C/EBP protein and gene. **(A)** Immunoblotting of C/EBPβ (C/EBPβ 35 kDa, LAP 32 kDa, and LIP 20 kDa) and C/EBPδ (30 kDa) from protein isolated from A-C/EBP protein- and gene-transfected cells. **(B)** Relative quantitative analysis of C/EBPβ, LAP, and LIP isoforms and C/EBPδ w.r.t actin gene. **(C)** Immunoblotting of adipocyte markers C/EBPα (42 kDa), PPARγ (53 and 57 kDa), and 422/aP2 (16 kDa) from protein- and gene-transfected cells. **(D)** Relative quantitative analysis of C/EBPα, PPARγ, and 422/aP2 w.r.t β-actin. ^*^Significantly different from the control group (*p* < 0.05). Values are expressed as mean ± SD; *n* = 3 independent experiments.

A-C/EBP protein was designed to inhibit the DNA binding of all C/EBP family members (Krylov et al., 1995). A transgenic mice overexpressing A-C/EBP protein showed reduced C/EBPβ expression but elevated C/EBPα level that suggests higher specificity of action *in vivo* (Won et al., 2007). Earlier, it was shown that the heterodimer between peptide inhibitor A-ZIP and its targeted bZIP is degraded by the proteolytic machinery of the cell (Won et al., 2007; Rozenberg et al., 2009). To determine the effect of A-C/EBP on the expression of C/EBPβ, total protein was isolated from gene- and protein-transfected cells. The relative intensities of bands were quantified with respect to β-actin (Figures 5B,C). C/EBPβ expression (C/EBPβ and LAP isoforms, 35 and 32 kDa) was found to be 36% lower in 2 h A-C/EBP protein samples and 38% lower in 48 h A-C/EBP gene samples (Figure 5A). In contrast, LIP form (20 kDa) was found to be upregulated in gene-transfected cells in 2 and 48 h samples. C/EBPβ was degraded within 2 h of A-C/EBP protein transfection but was again detected as A-C/EBP degrades after 8 h (Supplementary Figure 3D). The protein expression of C/EBPδ was not affected by either A-C/EBP gene or protein transfections (Figures 5A,B). The third member of the C/EBP family, i.e., C/EBPα (42 kDa), was downregulated in protein and 48 h gene-transfected samples (Figure 5C). The expression of 422/aP2 followed similar trend as that of C/EBPα (Figure 5C). For PPARγ, the decrease in expression in both protein and 48 h gene transfection was comparable (Figure 5C). Degradation of C/EBPβ in the early stages of differentiation led to a lower expression of adipocyte marker genes. With concomitant decreased mRNA expression, *PPARγ*, *C/EBPα*, and *422/aP2* exhibited lower protein expression (Figures 3A–C, 5C,D).

### A-C/EBP Treatment of Cells Led to Reduced Binding of TF at C/EBP Regulatory Elements in Adipocyte Marker Genes Except C/EBPα

**Figure 6:**
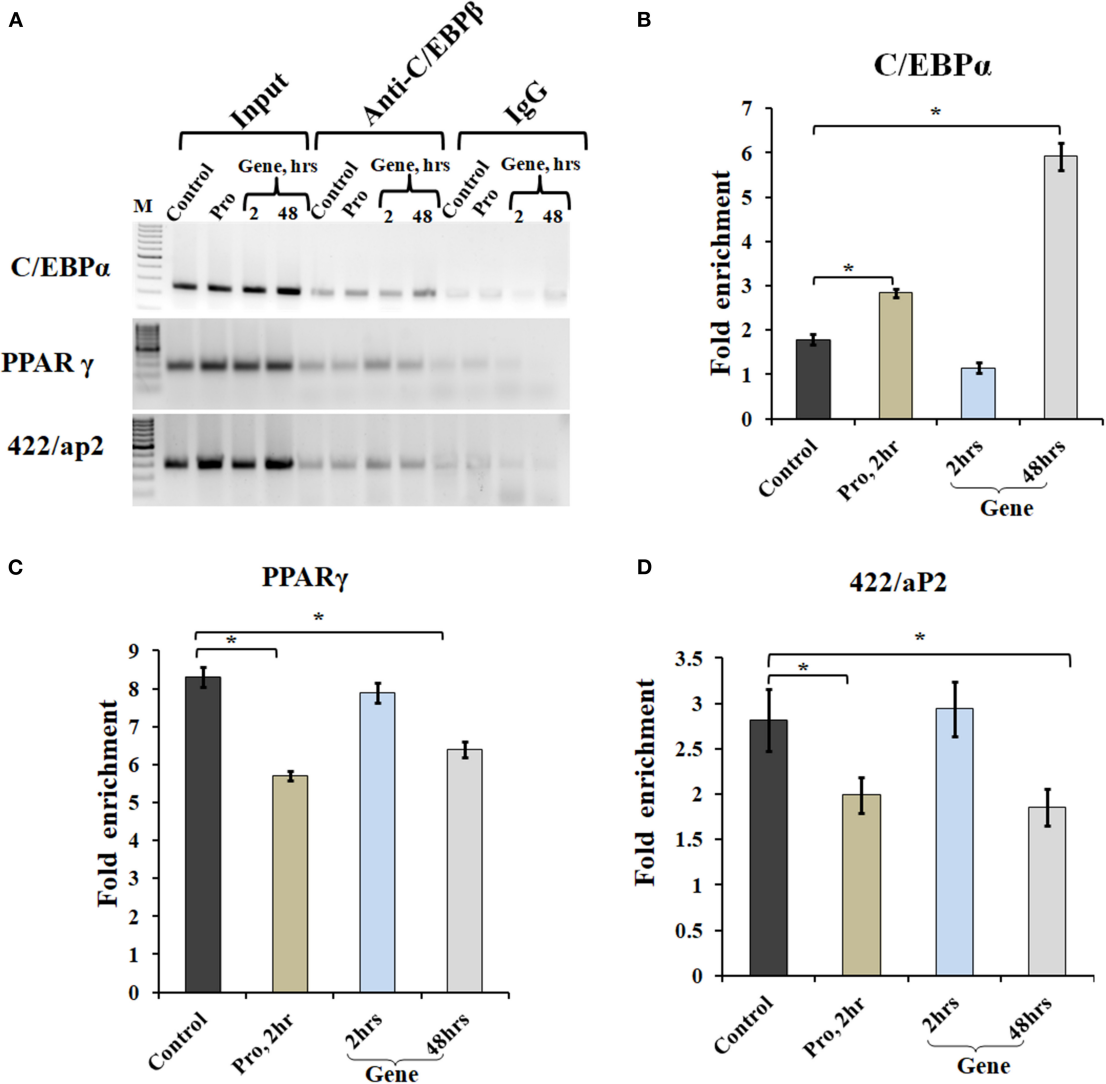
A-C/EBP transfection affects the binding of C/EBPβ to the promoter region of adipocyte marker genes. **(A)** Semi-quantitative PCR from ChIP samples showed binding of C/EBPβ to the C/EBP regulatory element in the promoter region of *C/EBPα*, *PPARγ*, and *422/aP2* in transfected cells. **(B–D)** Quantitative real-time PCR from ChIP samples of transfected cells with A-C/EBP protein and gene. Values are expressed as mean ± SD; *n* = 3 independent experiments. ^*^Significantly different from the control group (*p* < 0.05). Values are expressed as mean ± SD; *n* = 3 independent experiments.

Chromatin IP, followed by semi-quantitative PCR and qPCR, was performed for TF binding at promoter sites of adipocyte marker genes. Earlier studies showed that C/EBPβ acts as a transcriptional activator of both *C/EBPα* and *PPARγ* (MacDougald and Lane, 1995; Lane et al., 1999). After acquiring DNA-binding activity, C/EBPβ binds to regulatory elements in promoters of *C/EBPα*, *PPARγ*, and *422/aP2* genes (Tang et al., 2004). To determine C/EBPβ binding to the promoter region of these genes, ChIP assays were performed using cells transfected with A-C/EBP protein (2 h) and gene (2 and 48 h). Three days post-differentiation cells were cross-linked and immunoprecipitated with anti-C/EBPβ antibody, and PCR primers corresponding to adipocyte marker genes were used. Following A-C/EBP gene and protein transfections, reduced binding was observed for C/EBPβ at the promoter region of *PPARγ* and 422/aP2 in comparison with untransfected control and 2 h gene transfection samples (Figure 6A). Interestingly, the amplification of the promoter region of C/EBPα was enhanced in both protein and gene transfections. We construe this observation as enhanced binding of C/EBPα to its own promoter region, although enhanced binding did not lead to increased protein expression (Tang et al., 2004). The fold enrichment w.r.t. non-specific antibody agrees with semi-quantitative PCR results (Figures 6B–D).

## Discussion

Adipogenesis involving the conversion of fat-free preadipocytes to lipid-laden adipocytes is a time-dependent differentiation phenomenon that draws in many TFs during the process. This study was initiated with the assumption that the timely intervention and targeting TFs may inhibit preadipocytes differentiation, change cell fate, and reverse the process of differentiation. Triad of C/EBPβ, C/EBPα, and PPARγ play pivotal roles in preadipocyte differentiation. C/EBPβ is known to interact with other C/EBP family of TF including C/EBPα, C/EBPγ, and C/EBPζ. By using A-C/EBP that forms heterodimer with C/EBPβ and inhibits its nuclear localization and DNA-binding activity, we hoped to decipher the time-dependent inhibition of the differentiation process. In addition, it is imperative that A-C/EBP will also inhibit C/EBPβ interactions with its natural partners. In order to understand the time-dependent mechanism of the inhibition of adipogenesis by C/EBPs, we decided to study the direct and indirect partners of C/EBPβ that are known to regulate preadipocytes differentiation. In general, C/EBPβ binds C/EBP-binding site at the promoter regions of adipocyte marker genes, such as C/EBPα, PPARγ, and 422/aP2, thus regulating their expression.

Recently, protein transfection approaches are used to modulate the physiological processes in the cell. Earlier, on-target efficiency of the CRISPR/Cas9 nuclease system was evaluated by using pure Cas9 protein delivered directly into the cells, and its activity was compared with mRNA and gene transfections. The presence of Cas9 protein was observed within hours of transfection, whereas it took 24 h to express the Cas9 protein from DNA (Liang et al., 2015). Similarly, in this study, A-C/EBP protein signal peaked within 2 h of transfection, whereas it took 48 h for A-C/EBP plasmids to express the equivalent amount of protein (Figures 2A–D). A major advantage of using A-C/EBP is the formation of highly stable C/EBPβ**|**A-C/EBP heterodimer that is two magnitude stronger than C/EBPβ-DNA complex, thereby precluding the requirement of A-C/EBP overexpression (Krylov et al., 1995). Furthermore, A-C/EBP specifically impacts the adipogenic pathway as 3T3-L1 cells transfected with A-VBP, an inhibitor of VBP, aZIP TFs did not influence the differentiation process. Using A-C/EBP coding adenovirus, a previous study showed the prevention of translocation of C/EBPβ into the nucleus (Zhang et al., 2004b). Along similar lines, the expression level of C/EBPβ from A-C/EBP protein-transfected samples showed lower protein level after 2 h, whereas A-C/EBP plasmid samples showed reduced C/EBPβ signals only after 48 h of initiation of differentiation (Figures 5A,B). Degradation of C/EBPβ in the early stages of differentiation led to the lower expression of *PPARγ*, *C/EBPα*, 422/aP2, and other adipocyte marker genes (Figures 5C,D).

We further analyzed the expression profiles of known C/EBP interacting partners. Stat5a is known to co-localize with C/EBPβ, whereas CREB binds to the promoter region of *C/EBPβ*, and GATA2/3 form protein complexes with C/EBPβ/α (Zhang et al., 2004a; Tong et al., 2005; Kang et al., 2013). In addition, STAT5a and CREB co-express with C/EBPβ, i.e., after an hour of induction, whereas GATA2/3 is expressed at the onset of adipogenesis (Reusch et al., 2000; Tong et al., 2005; Kang et al., 2013). With the degradation of C/EBPβ at the early stage of differentiation, there is a concomitant decrease in the expression of its direct interacting partners as indicated by lower expression of *Stat5a* and *Creb*. The observed higher *GATA2* expression in A-C/EBP protein-transfected cells is due to the sequestering of C/EBPβ by A-C/EBP. GATA2 is known to bind C/EBPβ/α. In the absence of C/EBPβ, it binds to C/EBPα leading to the suppression of adipogenesis in protein-transfected cells. In A-C/EBP plasmids (6 h) samples, no change in expression was observed for GATA2/3, whereas in 48 h, GATA3 mRNA was upregulated without any change in the GATA2 level. The observed expression profiles of GATA2/3 need further investigations. This observation reinforces the advantages of using proteins and peptides to study the spatial and temporal gene regulation in complex pathways, such as adipogenesis. *VEGF, FoxC2, TCF7L1*, and *KLF2* are not known to interact or have indirect interaction with C/EBPβ, and their upregulation in A-C/EBP gene but not in A-C/EBP protein-transfected samples suggests to the non-specific interactions of A-C/EBP protein at higher and persistent expression.

C/EBPβ is required for MCE during the adipogenesis process, and its inhibition affects the normal differentiation process in preadipocyte (Tang et al., 2003). We observed inhibitory effect of A-C/EBP on MCE as cells treated with A-C/EBP protein (2 h) and gene (48 h) showed higher percentage of cells in the G0–G1 phase than untreated and A-C/EBP gene- (2 h) transfected cells. In addition, p27 restrains Cdk2/CyclinA from crossing the G1–S checkpoint, and its downregulation is necessary for cells to cross the G1–S checkpoint (Zhang et al., 2004b). We observed that A-C/EBP protein- and gene- (48 h) treated cells were trapped in the G0–G1 phase as p27 was not downregulated and cells were unable to cross the checkpoints.

**Figure 7:**
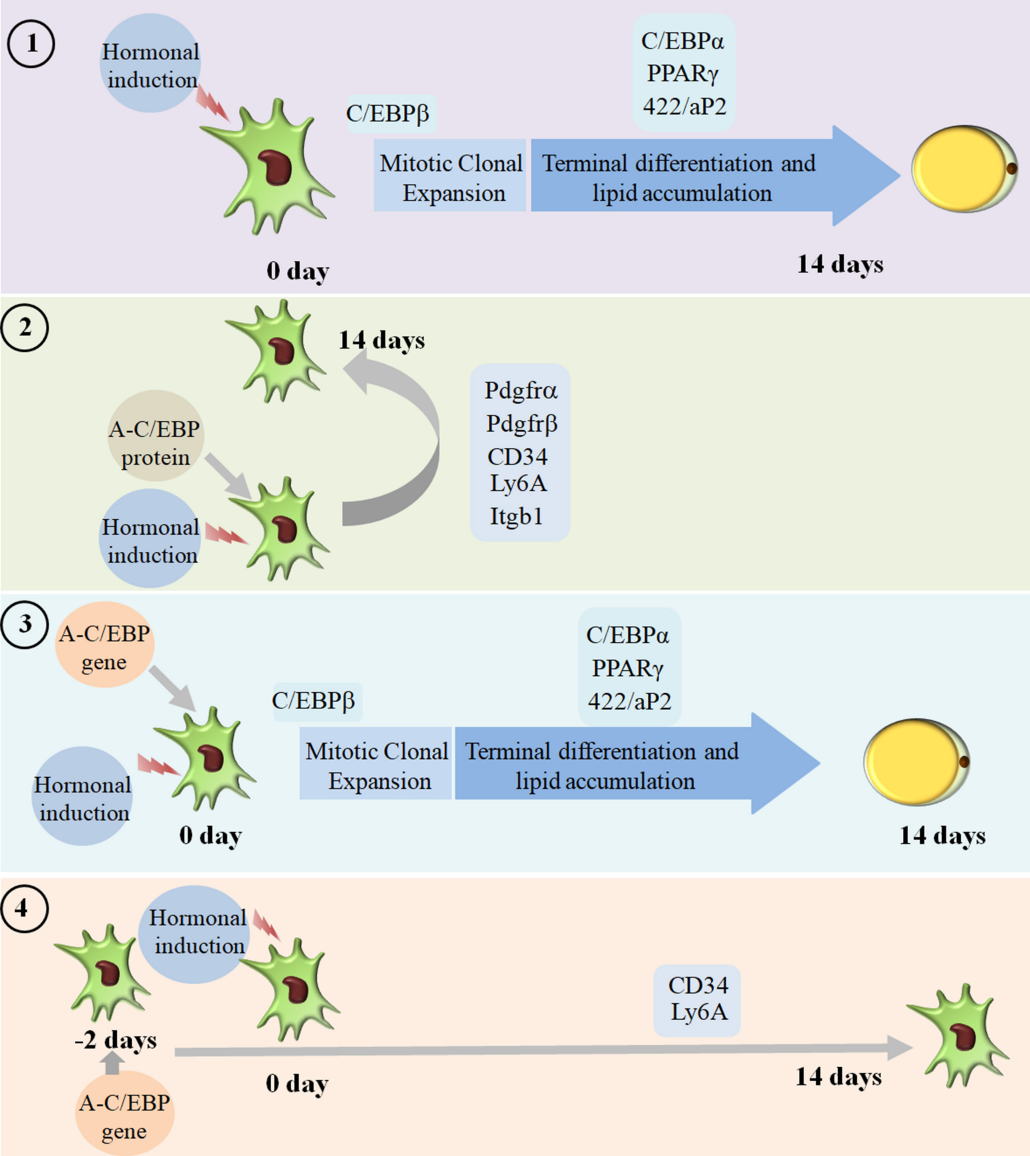
Summary of the studies showcasing the results obtained when 3T3-L1 cells were transfected with A-C/EBP protein and gene. Four experimental conditions are depicted here. **(1)** Normal differentiation protocol was followed. 0 day defines the day when cells were exposed to MDI cocktail of hormones. The differentiation process takes 14 days that involves MCE and terminal differentiation. Process is marked by transformation of flaccid preadipocytes into lipid-laden mature adipocytes. Only the master regulators of adipogenesis, e.g., *C/EBPβ*, *C/EBPα*, *PPARγ*, and *422/aP2*, is shown. **(2)** Cells were transfected with A-C/EBP protein for 2 h and induced to differentiate. Following the differentiation protocol, the cells were tested for phenotype and analyzed for molecular markers. The cells failed to differentiate as determined by Oil Red O staining. The cells transfected with A-C/EBP protein still express preadipocyte markers 5 days post-differentiation. Five preadipocyte markers were upregulated; among them, *Pdgfrα* and *Pdgfr*β are responsible for white adipocyte tissue (WAT) to beige adipocyte cell fate determination. **(3)** The cells were transfected with A-C/EBP gene for 2 h and induced to differentiate. After 14 days, preadipocytes convert into lipid-laden adipocytes with the expression of adipocyte marker genes. **(4)** The cells were transfected with A-C/EBP gene for 48 h and induced to differentiate. After 14 days, the cells were stained with Oil Red O, and the expression profiles of adipocyte differentiation markers were analyzed. Unlike protein samples, only two *CD34* and *Ly6A* preadipocyte markers were expressed.

We went on to explore if we could revert cell fate and de-differentiate adipose cells. Figure 7 shows the experimental design and summary results obtained when 3T3-L1 cells were transfected with A-C/EBP protein and gene. De-differentiation of mature adipocyte during lactation into preadipocyte precursor cells showed the expression of common preadipocyte markers, namely, *Pdgfrβ*, *Pdgfrα*, *Ly6A* (*Sca1*), *CD34*, and *Itgb1* (fibronectin receptor) (Wang et al., 2018). Gene expression analysis of common preadipocyte markers from cells 5 days post-differentiation has shown higher expression of all five genes in A-C/EBP-protein transfected cells. In contrast, we observed the non-significant difference in the expression of *Pdgfrβ*, *Pdgfrα*, and *Itgb1* but higher expression of *Ly6A* and *CD34* in gene- (48 h) transfected cells. *Ly6A, CD34*, and *Itgb1* are the common mesenchymal stem cell markers, and the signaling balance between *Pdgfrβ/α* regulates the commitment of adipose progenitors to either white or beige adipose tissues (Cawthorn et al., 2012; Gao et al., 2018). Earlier studies have shown the inhibitory role of *Pdgfrα* during adipogenesis as its activation leads to the inhibition of adipogenesis (Sun et al., 2017; Haider et al., 2018). In addition, mosaic deletion of both *Pdgfrα* and *Pdgfrβ* enhances the adipogenesis process as these act as negative regulator of adipogenesis (Sun et al., 2020). Downregulation and degradation of Pdgfrs within 6 h of hormonal induction have been observed in 3T3-L1 cells (Vaziri and Faller, 1996). C/EBPβ is reported to transcriptionally repress *Pdgfrα* in vascular smooth muscle cells (VSMCs) (Kitami et al., 1999). Akin to VSMCs, our results show that C/EBPβ inhibition by A-C/EBP protein upregulates *Pdgfrα/β* after hormonal induction, thus inhibiting the conversion of preadipocytes to adipocytes. Our findings suggest that the higher expression of common preadipocyte markers is due to the non-conversion of the cells into adipocytes after hormonal induction. In gene transfection studies, the late expression of A-C/EBP protein though inhibits the differentiation process but did not induce all preadipocyte marker genes. A-C/EBP gene-transfected cells show similar morphology as that of protein-transfected cells since the late expression of A-C/EBP leads to the suppression of C/EBPβ that further inhibits the expression of C/EBPα and PPARγ, two master regulators of terminal adipocyte formation.

A strategy to introduce exogenous protein directly to the mammalian cells may control the fate of differentiating cells and may be applied to study signal transduction that involved the co-ordinated and timely expression of a number of TFs. Elaborate and quantitative proteomic studies will decipher the absolute and relative expression levels of preadipocyte markers that decide the cell fate.

## Data Availability Statement

The raw data supporting the conclusions of this article will be made available by the authors, without undue reservation.

## Ethics Statement

Studies involving animal subjects: no animal studies are presented in this manuscript. Studies involving human subjects: no human studies are presented in this manuscript. Inclusion of identifiable human data: no potentially identifiable human images or data is presented in this study.

## Author Contributions

NS and VR designed the study and analyzed the data and wrote the manuscript. NS conducted the experiments with help from RK, KS, HP, DC, PJ, and AA. Confocal microscopy experiments were performed with help from BY. CV provided the reagents. All authors reviewed the results and approved the final version of the manuscript.

## Conflict of Interest

The authors declare that the research was conducted in the absence of any commercial or financial relationships that could be construed as a potential conflict of interest.
