# Supplementary figures and images for: Transient Delivery of A-C/EBP Protein Perturbs Differentiation of 3T3-L1 Cells and Induces Preadipocyte Marker Genes
Source: Front Mol Biosci. 2021 Jan 25;7:603168. doi: 10.3389/fmolb.2020.603168 (PMC7868408; doi:10.3389/fmolb.2020.603168)

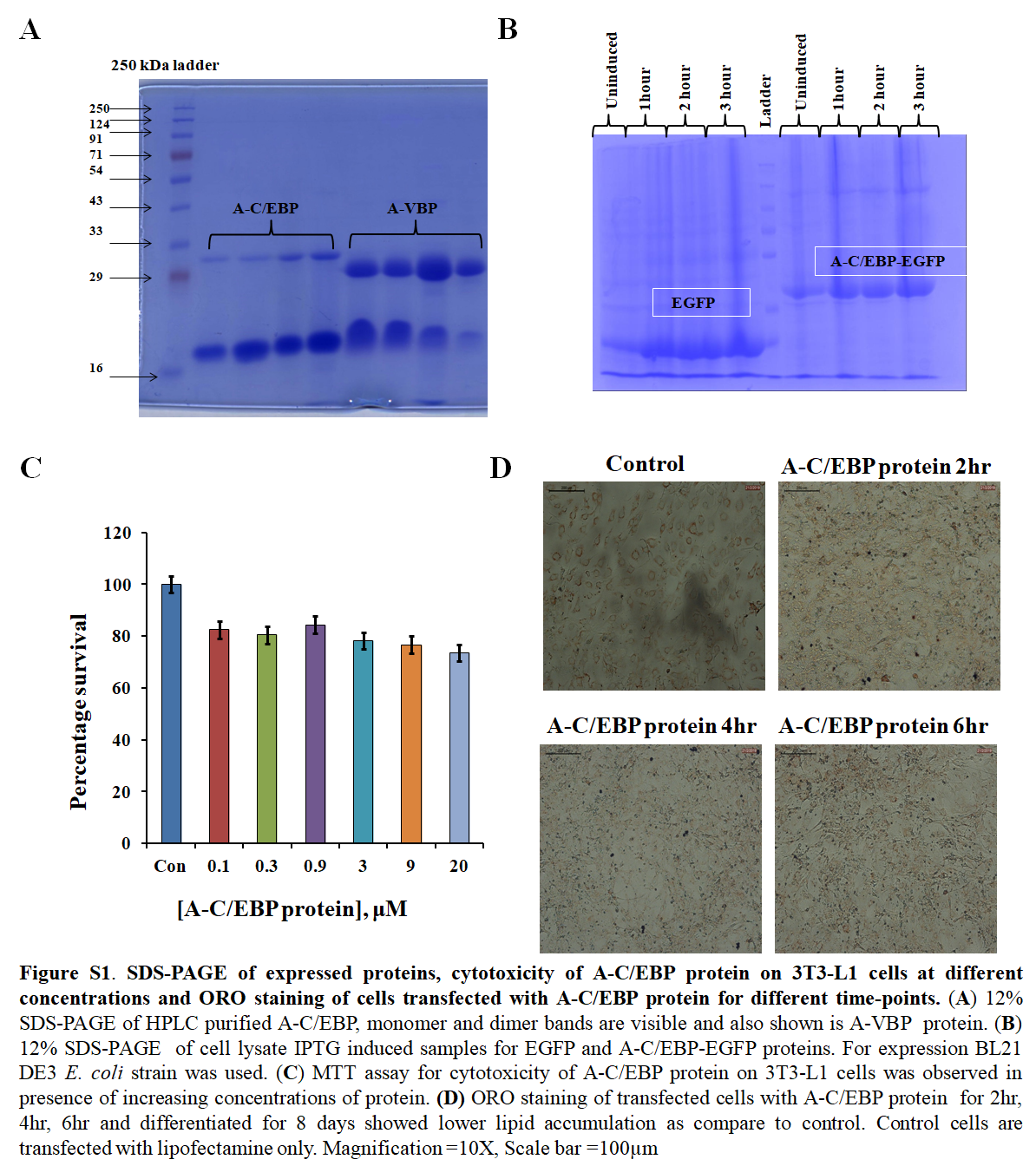

Supplement: Supplementary file 2 [file Image_1.TIF]

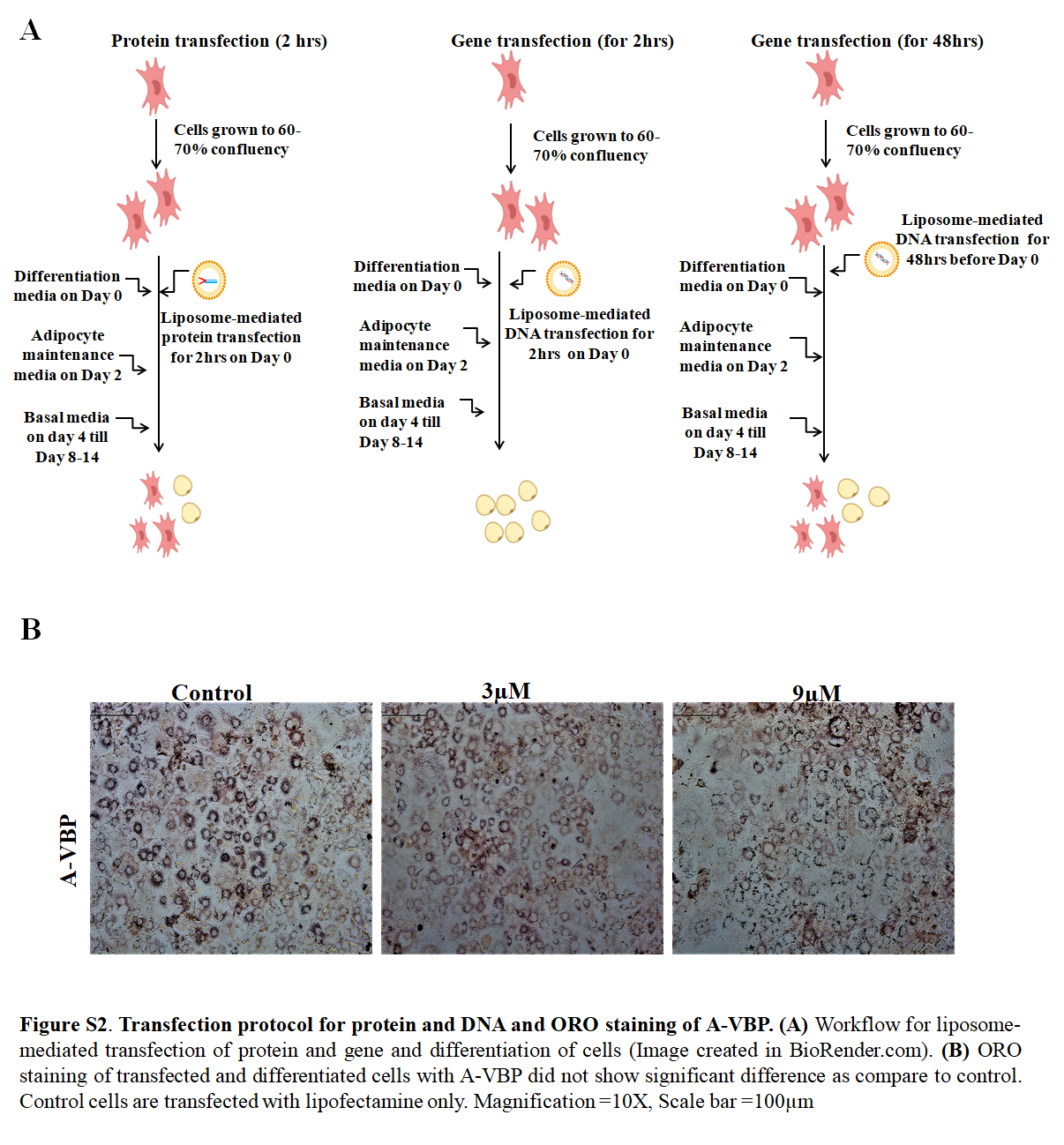

Supplement: Supplementary file 3 [file Image_2.TIF]

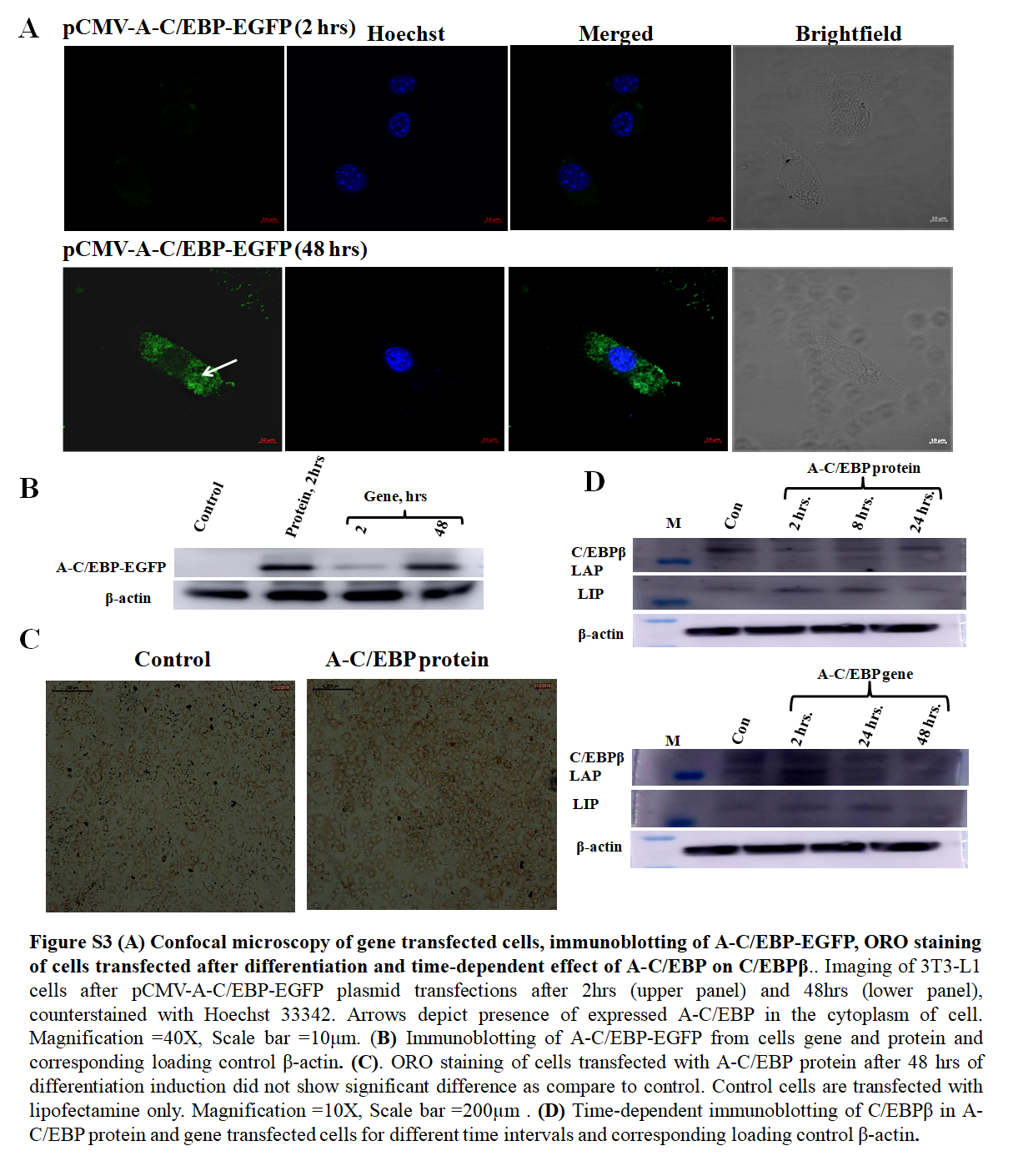

Supplement: Supplementary file 4 [file Image_3.TIF]

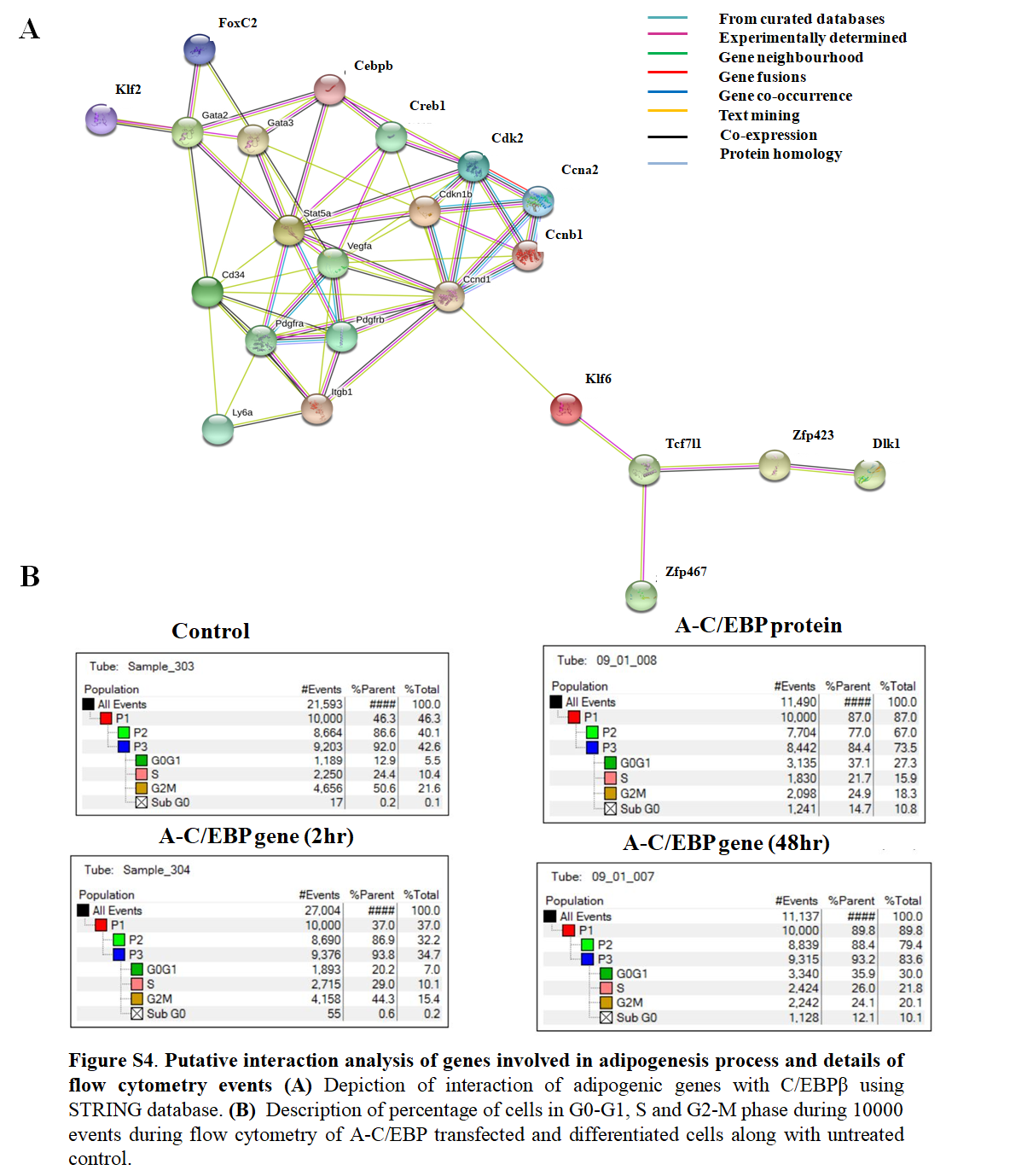

Supplement: Supplementary file 5 [file Image_4.TIF]
